# High-sensitivity troponin T levels before and after cardiac surgery and the 30-day mortality: a retrospective cohort study

**DOI:** 10.3389/fcvm.2023.1276035

**Published:** 2023-11-30

**Authors:** Jian-Wei Liang, Min Zhou, Yong-Qiang Jin, Ting-Ting Li, Jiang-Ping Wen

**Affiliations:** ^1^ Laboratory Medicine Department, Tsinghua University First Hospital, Beijing, China; ^2^ Obstetrics and Gynecology Department, Tsinghua University First Hospital, Beijing, China; ^3^ Department of Medical Laboratory, Tianjin Medical University, Tianjin, China

**Keywords:** heart surgery, myocardial injury, cardiac troponin T, 30-day mortality rate, coronary artery bypass grafting, aortic valve replacement or repair

## Abstract

**Background:**

The suggested threshold level of cardiac troponin T elevation after cardiac surgery is not very clear, and the values recommended by various guidelines and literature reports are quite different.

**Methods:**

In this retrospective cohort study, we collected clinical data of patients who underwent heart surgery at Tsinghua University First Hospital between January 2015 and December 2022. Using the high-sensitivity cardiac troponin T levels (reference upper limit: 14 ng/L) measured at 1–3 days postoperation, the relationship between the cardiac troponin T level and the 30-day mortality risk was evaluated using Cox regression analysis.

**Results:**

Among the 3,128 patients included in this study, the types of operations mainly consisted of coronary artery bypass graft (CABG, 1,164, 37.2%), aortic valve replacement (AVR, 735, 23.5%), and other cardiac operations (1,229, 39.3%). Within 30 days postoperation, 57 patients (1.8%) died and 72 patients (2.3%) developed major vascular complications. In patients undergoing CABG or AVR, the cardiac troponin T threshold level measured within one day postoperation related to an increased 30-day mortality was determined to be 3,012 ng/L (95% CI: 1,435–3,578 ng/L), which is 218 times higher than the reference upper limit. In patients undergoing other cardiac operations, this threshold was 5,876 ng/L (95% CI: 2,458–8,119 ng/L), which is 420 times higher than the reference upper limit.

**Conclusion:**

The high-sensitivity cardiac troponin T level associated with an increased 30-day mortality risk after cardiac surgery is significantly higher than the current recommendations for defining clinically important perioperative myocardial injury.

## Introduction

The 2021 White Paper on the Survey of Cardiac Surgery and Cardiopulmonary Bypass Data in China, which was released at the theme meeting of the 2022 China Cardiopulmonary Bypass Academic Annual Conference, reported that there were 278,056 cardiac great vessel operations and 176,496 cardiopulmonary bypass operations in China in 2021 (1). Although heart surgery remains the best hope for improving the physical condition and prolonging the life of heart patients, the associated complications cannot be ignored (2, 3). While hemorrhage, craniocerebral injury, and pleural effusion are the most common postoperative complications, myocardial injury is also a complication that warrants medical attention (4). To clinically evaluate the existence and severity of myocardial injury, the detection of cardiac troponin or creatine kinase MB in serum/plasma is the most commonly used approach. The level of cardiac troponin or creatine kinase MB has been reported to be positively correlated with the mortality risk (5–7).

In recent years, creatine kinase MB has become a widely used marker for judging myocardial injury. However, accumulating studies and several consensus statements have upheld cardiac troponin as the first-choice marker for myocardial injury (8, 9). Although the absolute threshold value of myocardial injury markers after cardiac surgery remains controversial, there is a clear relationship between cardiac troponin elevation and myocardial infarction (MI) after various cardiac operations; as per expert opinions, a value 70 times higher than the reference upper limit can indicate myocardial injury during the perioperative period (1, 8, 10). Nevertheless, the relationship between the postoperative increase in the cardiac troponin T level and the risk of adverse events or even death after cardiac surgery is not particularly clear (11). In this study, we retrospectively analyzed the relevant data before and after cardiac surgery to clarify the relationship between the change in the high-sensitivity cardiac troponin T level and the risk of adverse events after cardiac surgery.

## Materials and methods

### Patients and inclusion and exclusion criteria

We collected data from patients who underwent cardiac surgery at The First Affiliated Hospital of Tsinghua University, China, between January 2015 and December 2022. Patients whose blood high-sensitivity cardiac troponin T levels were quantified at 48 h before and 72 h after heart surgery were selected. Patients whose preoperative findings showed any evidence of MI (e.g., high-sensitivity cardiac troponin T level above the upper limit of the reference range and myocardial ischemia confirmed by electrocardiogram) were excluded.

### Collection process

This study received ethical approval from the Ethics Committee of Tsinghua University First Hospital, and informed consent was waived due to the retrospective character of the present study. We obtained the data on risk factors of potential cardiac surgery complications in these patients by reviewing the patients’ historical medical records, laboratory tests, and other reports. Given that this was a retrospective study, some patients’ data records regarding some parameters were inevitably missing. Missing components for the calculation of the EuroSCORE II were imputed with the use of multiple imputation. All included risk factors were obtained from the 18 variables mentioned in EuroSCORE II (12) (e.g., age, sex, and other clinical data), and the mortality risk of patients after cardiac surgery was evaluated by the weight of the procedure. For inclusion in this study, the Roche HS-TnT reagent must have been used to determine the patients’ cardiac troponin T levels within 48 h before cardiac surgery and at 24 h, 48 h, and 72 h after surgery.

Death within 30 days postoperation was considered as the main outcome. Secondary outcomes mainly included complications within 30 days postoperation, postoperative stay in the intensive care unit, MI, and other surgery-related adverse events.

### Statistical analysis

SPSS 26.0 software was used for all statistical analyses. We included 18 predictive variables (all from EuroSCORE II) in the Cox proportional hazard regression model of the 30-day mortality, and the missing data were interpolated by multiple interpolation. The relationship between the high-sensitivity cardiac troponin T level within one day postoperation and the 30-day mortality was evaluated by Cox statistical analysis. Pearson correlation was used to analyze the troponin T thresholds.

## Results

### Baseline data of the cohort population

Among the 3,128 patients finally included in this study (Table 1), the median level of high-sensitivity cardiac troponin T measured within 48 h before the operation was 7 ng/L (interquartile range: 3–15 ng/L). The operation types mainly included simple valve replacement [coronary artery bypass graft, (CABG), 1,164 cases, 37.2%], aortic valve replacement or repair (AVR, 735 cases, 23.5%), and other heart operations (1,229 cases, 39.3%). The average EuroSCORE II was 2.8%.

**Table 1:** Baseline data of the cohort population.

Variable	Total (*N* = 3128)
Mean age (±standard deviation), years^a^	58.2 ± 11.3
Male sex, no. (%)^a^	2314 (74.0%)
Clinical history	
Myocardial injury, no. (%)	2452 (78.4%)
Myocardial infarction occurring within three months before surgery, no. (%)^a^	524 (16.8%)
Smoke, no. (%)	1652 (52.8)
Stroke, no. (%)	102 (3.3%)
Peripheral arterial disease, no. (%)^a^	157 (5.0%)
Hypertension, no. (%)	2014 (64.3%)
Heart failure, no. (%)	428 (13.7%)
New york heart association cardiac function classification, no. (%)^a^	
II	204 (6.3%)
III	174 (5.6%)
IV	65 (2.1%)
History of cardiac surgery, no. (%)^a^	88 (2.8%)
Chronic obstructive pulmonary disease, no. (%)^a^	156 (5.0%)
Diabetes, no. (%)	456 (14.6%)

^a^ This variable was selected based on the European System for Cardiac Operative Risk Evaluation II (EuroSCORE II).

### Cardiac troponin T level

The median cardiac troponin T level in patients with high sensitivity was 2,115 ng/L (interquartile range, 857–4,356 ng/L) within 3 days postoperation. Blood tests to quantify the cardiac troponin T levels were conducted daily for some patients and only once or twice for others, depending on the doctors’ judgment of how often the testing was required.

**Figure 1:**
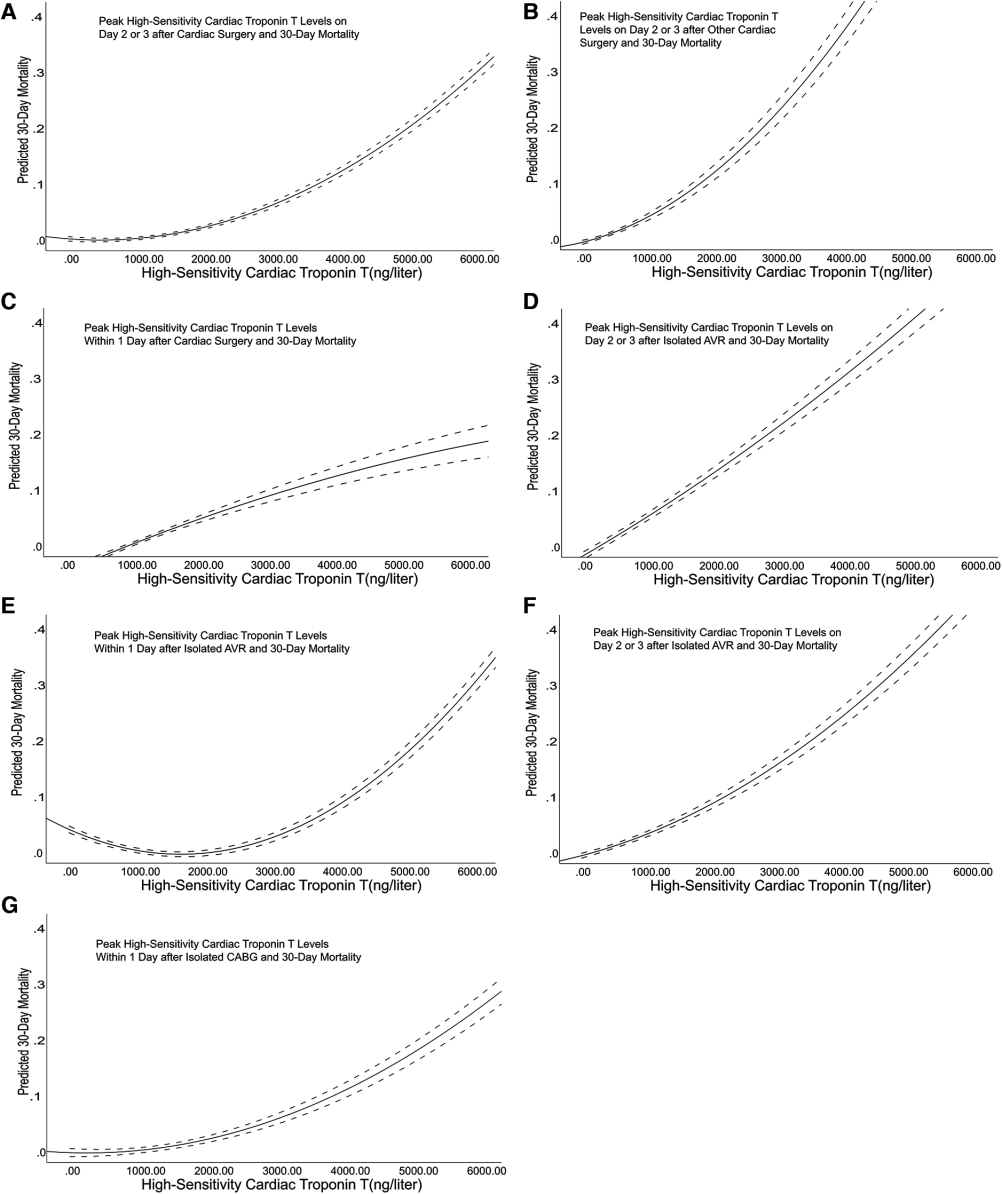
Correlation diagram between the cardiac troponin T level and the outcome. The dotted lines represent the 95% confidence interval of the unadjusted relationship between the cardiac troponin T measurement value of the peak hypersensitive myocardium and the baseline. The graph depicts this relationship at 2 or 3 days after surgery, including cardiac surgery (Panel A), other cardiac surgery without AVR and CABG (Panel B), AVR (Panel D), and CABG (Panel F). The graph also depicts this relationship on the first day after surgery, including cardiac surgery (Panel C), AVR (Panel E), and CABG (Panel G).

The cardiac troponin T levels in 2,654 patients were collected on the first day after cardiac surgery. Among them, 2,537 patients (95.6%) had cardiac troponin T levels greater than 140 ng/L (≥10 times the upper reference limit), and 2,357 patients (88.8%) had cardiac troponin T levels greater than 490 ng/L (≥35 times the upper reference limit). Within 30 days postoperation, 57 patients (1.8%) died and 72 patients (2.3%) developed major vascular complications. We adjusted for the following factors: the New York Heart Association (NYHA) classification for dyspnea, insulin-dependent diabetes mellitus, extracardiac arthritis, poor mobility, previous cardiac surgery, renal dysfunction, active endocarditis, critical preoperational state, left ventricular (LV) function or left ventricular ejection fraction (LVEF), urgency of procedure, recent MI (within 90 days before the operation), and weight of the procedure. A diagram showing the correlation between the cardiac troponin T level and the 30-day outcome is shown in Figure 1.

**Figure 2:**
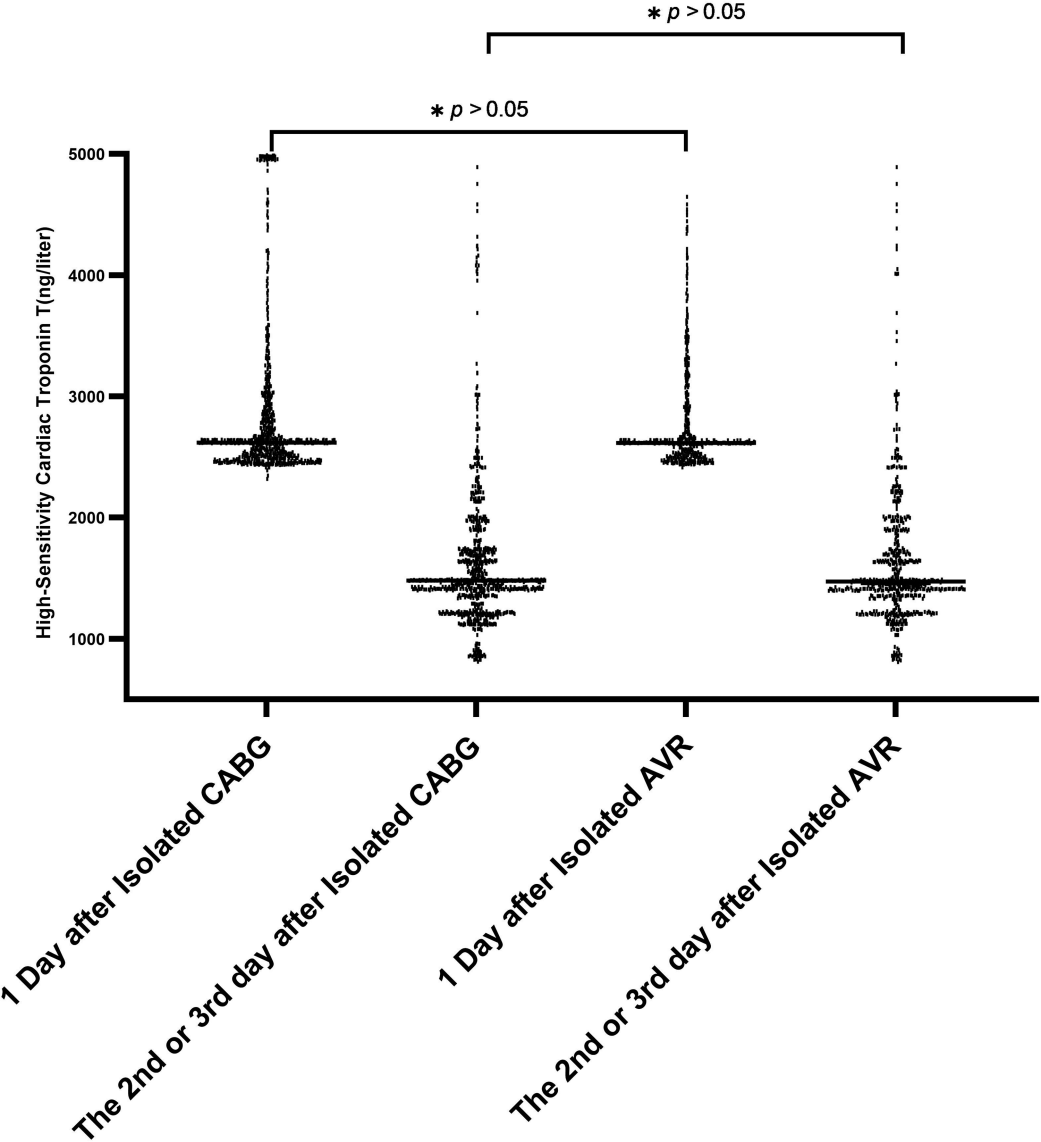
Cardiac troponin T level after cardiac surgery.

The results obtained in our study showed no significant difference in the levels of high-sensitivity troponin measured on the first, second, and third days after cardiac surgery between the CABG group and the AVR group. Therefore, we merged the two groups. The results are shown in Figure 2. In patients receiving CABG or AVR alone, the lowest high-sensitivity cardiac troponin T level measured within one day postoperation that was related to the adjusted hazard ratio of the 30-day mortality was 3,012 ng/L (95% CI: 1,435–3,578 ng/L), which is 215 times higher than the reference upper limit. On the second or third day postoperation, the value was 758 ng/L (95% CI: 354–2019 ng/L), which is 54 times higher than the reference upper limit. Among patients who underwent other cardiac operations, the lowest high-sensitivity cardiac troponin T level measured one day postoperation that was related to the 30-day mortality risk was 5,876 ng/L (95% CI: 2,458–8,119 ng/L), which is 420 times higher than the reference upper limit. On the second or third day postoperation, the threshold was 987 ng/L (95% CI: 548–2,542 ng/L), which is 70 times higher than the reference upper limit.

### Fold increase of the cardiac troponin T level compared with the baseline level

**Figure 3:**
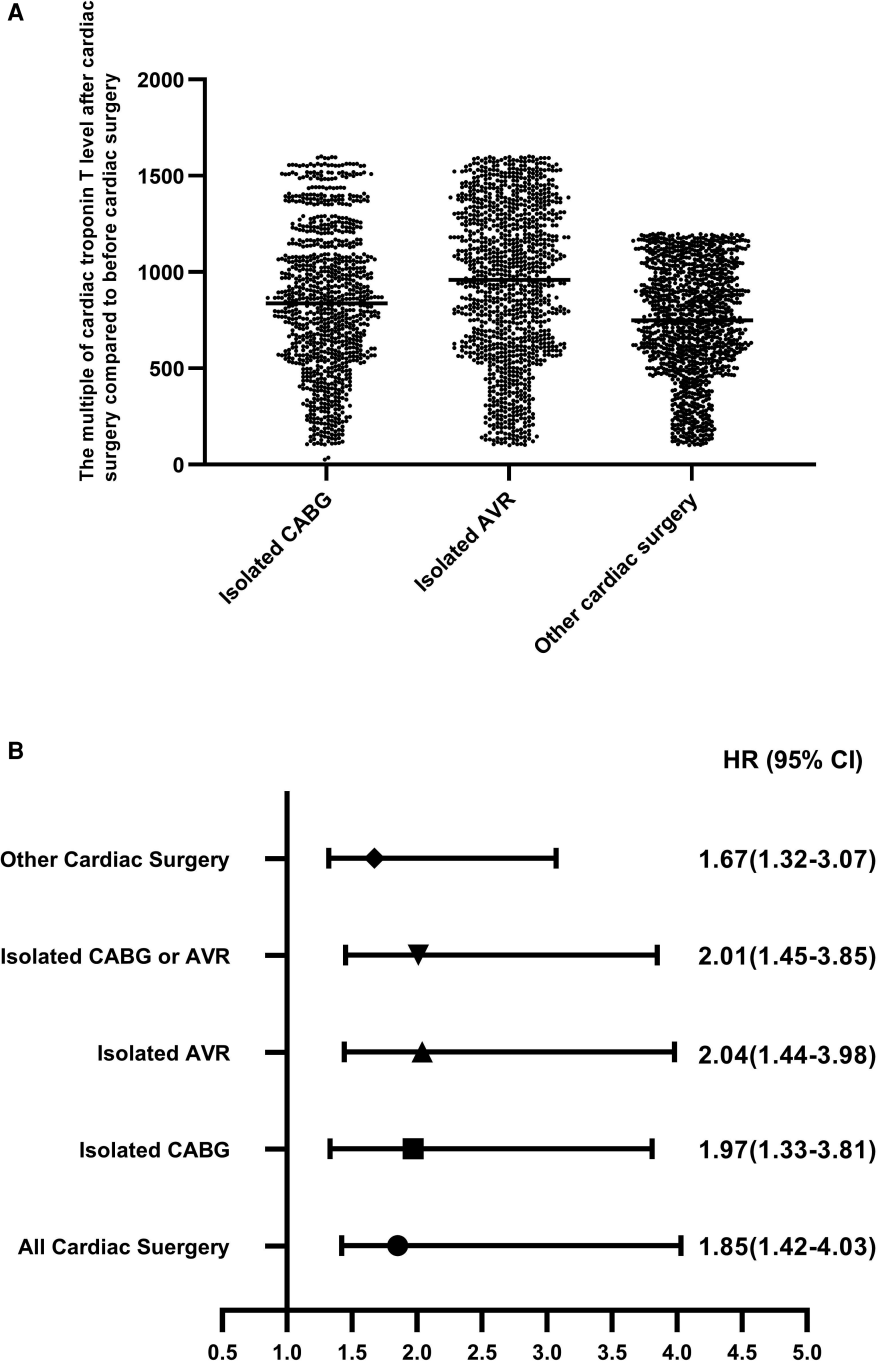
The fold increase of the cardiac troponin T level and the risk of 30-day mortality (every 500-fold increase) after the operation compared with before the operation. The fold increase of cardiac troponin T levels after isolated AVR, isolated CABG, and other cardiac surgery compared with before the operation (Panel A). The fold increase of cardiac troponin T level and the risk of 30-day mortality (every 500-fold increase) after the operation compared with before the operation (Panel B).

In our study, the postoperative level of cardiac troponin T was higher than the preoperative level. We adjusted for the following factors: age, sex, extracardiac artistry, pulmonary disease, neurological or musculoskeletal dysfunction, previous cardiac surgery, serum creatinine level, active endocarditis, critical preoperational state, NYHA class, LVEF, recent MI within 90 days before the operation, systolic pulmonary pressure, and urgency of procedure. The fold increases of cardiac troponin T levels after CABG, AVR, or other cardiac surgery were 243 (95% CI: 176–782), 262 (95% CI: 168–967), and 212 (95% CI: 114–852), respectively. The relationship between the fold increase of the cardiac troponin T level postoperation and the risk of 30-day mortality is shown in Figure 3.

## Discussion

In our study, the mortality rate was higher in women than in men. Extracardiac artery disease, lung disease, and the preoperative critical state can all have an impact on mortality. Meanwhile, as the NYHA level increases, the risk of death increases accordingly. The inconvenience caused by various functional factors and previous cardiac surgeries can also impact the final mortality rate. The many factors that contribute to the increased final mortality rate have been confirmed multiple times; thus, we focused on the troponin T levels in this study. We retrospectively analyzed the relationship between patients’ postoperative high-sensitivity cardiac troponin T level and their clinical outcome. Through correlation analyses between the high-sensitivity cardiac troponin T levels and the 30-day mortality risk, we finally obtained the threshold corresponding to our data. The high-sensitivity cardiac troponin T level thresholds were identified as 3,012 ng/L within one day of CABG or AVR and as 758 ng/L on the second or third day postoperation. In patients undergoing other operations, the threshold of high-sensitivity cardiac troponin T within one day postoperation was relatively high, reaching 5,876 ng/L, and the threshold on the second or third day was 987 ng/L. Although the threshold recommended in the known consensus by Garcia et al. is markedly lower than that identified in our research (11), their threshold is an important basis for the clinical diagnosis of postoperative MI or other myocardial injury (13–15).

Previous studies (16–19) have shown that in cases of cardiac surgery, the postoperative cardiac troponin T level is clearly associated with an increased 30-day mortality risk. Most studies have shown that the lowest cardiac troponin T threshold related to an increased 30-day mortality risk is 30–100 times higher than the upper limit of the reference range. The lowest cardiac troponin T threshold within one day postoperation was higher in our study than in previous reports.

At the same time, the data observed in our study show that the fold increase over the baseline level observed for the high-sensitivity cardiac troponin T level measured after cardiac surgery can also be an important basis for clinical evaluation of the 30-day mortality risk. In this study, the minimum threshold value of the preoperative/postoperative ratio of high-sensitivity cardiac troponin T related to the 30-day mortality risk after cardiac surgery was 1,057, and patients exceeding this ratio need more attention from clinicians.

Importantly, the 30-day mortality risk is not the only direct harm caused by myocardial injury, but it is also related to the abilities of the operators and postoperative nurses (19). Additionally, the patient's own medical compliance will also play an important role (15). In any case, it is necessary to detect high-sensitivity cardiac troponin T in a timely manner before and after cardiac surgery, which is obviously helpful for clinicians to fully evaluate the mortality risk of patients. However, a low threshold leads to not only an increased postoperative mortality risk and economic burden for the patient as well as an increased clinical work intensity of healthcare professionals but also an increased distrust of patients in their doctors. Considering these factors, we suggest that the minimum threshold level of high-sensitivity cardiac troponin T for the 30-day mortality risk after cardiac surgery should be raised. The minimum threshold levels of high-sensitivity cardiac troponin T within 1 day and 2–3 days after CABG or AVR should be set to 3,000 ng/L and 700 ng/L, respectively, and the minimum threshold levels of high-sensitivity cardiac troponin T within 1 day and 2–3 days after other cardiac operations should be set to 5,800 ng/L and 980 ng/L, respectively. Besides focusing on the above measures, doctors should also closely monitor any changes in the high-sensitivity cardiac troponin T levels of the patients. When the postoperative/preoperative ratio exceeds 1,000, prompt measures to prevent or reduce the occurrence of adverse outcomes should be taken.

Our research has some limitations that must be addressed. Given the retrospective design of this study, we could not always obtain the data we needed from the clinical records. In addition, the integrity and findings of this study need to be confirmed by a prospective study. Moreover, our study did not consider long-term outcomes, such as heart failure, reoperation, and heart-related deaths occurring within 30 days postoperation. As the association between myocardial injury caused by heart surgery and the long-term results remains to be explored, data from a longer follow-up period are needed to further confirm the lowest threshold of high-sensitivity cardiac troponin T, which may be lower than our current findings. Reasons explaining the increase of the high-sensitivity cardiac troponin T levels in the present study remain unclear. Although the majority of the increase can be attributed to the heart surgery itself, the increase may still be influenced by MI in patients. Even though our study distinguishes between CABG and AVR, it does not more elaborately distinguish among heart surgeries and related factors, including the urgency of the surgery, the qualifications of the operator, and the nursing and treatment methods during and after the surgery. Therefore, a rigorous prospective study is warranted to further verify the reliability of our findings and conclusions.

## Conclusion

The high-sensitivity cardiac troponin T level associated with an increased 30-day mortality risk after cardiac surgery is significantly higher than the current recommendations for defining clinically important perioperative myocardial injury.

## Data Availability

The raw data supporting the conclusions of this article will be made available by the authors, without undue reservation.
